# Influence of oxygen tension on myocardial performance. Evaluation by tissue Doppler imaging

**DOI:** 10.1186/1476-7120-2-22

**Published:** 2004-11-02

**Authors:** Ole Frøbert, Jacob Moesgaard, Egon Toft, Steen Hvitfeldt Poulsen, Peter Søgaard

**Affiliations:** 1 Department of Cardiology, Center for Cardiovascular Research, Aalborg Hospital, Aarhus University Hospital, Denmark; 2 Institute of Pharmacology, University of Aarhus, Denmark; 3 Center for Model-based Medical Decision Support Systems, Department of Health Science and Technology, Aalborg University, Aalborg, Denmark; 4 Skejby University Hospital, Aarhus, Denmark

## Abstract

**Background:**

Low O_2 _tension dilates coronary arteries and high O_2 _tension is a coronary vasoconstrictor but reports on O_2_-dependent effects on ventricular performance diverge. Yet oxygen supplementation remains first line treatment in cardiovascular disease. We hypothesized that hypoxia improves and hyperoxia worsens myocardial performance.

**Methods:**

Seven male volunteers (mean age 38 ± 3 years) were examined with echocardiography at respiratory equilibrium during: 1) normoxia (≈21% O_2_, 79% N_2_), 2) while inhaling a hypoxic gas mixture (≈11% O_2_, 89% N_2_), and 3) while inhaling 100% O_2_. Tissue Doppler recordings were acquired in the apical 4-chamber, 2-chamber, and long-axis views. Strain rate and tissue tracking displacement analyses were carried out in each segment of the 16-segment left ventricular model and in the basal, middle and apical portions of the right ventricle.

**Results:**

Heart rate increased with hypoxia (68 ± 4 bpm at normoxia vs. 79 ± 5 bpm, P < 0.001) and decreased with hyperoxia (59 ± 5 bpm, P < 0.001 vs. normoxia). Hypoxia increased strain rate in four left ventricular segments and global systolic contraction amplitude was increased (normoxia: 9.76 ± 0.41 vs hypoxia: 10.87 ± 0.42, P < 0.001). Tissue tracking displacement was reduced in the right ventricular segments and tricuspid regurgitation increased with hypoxia (7.5 ± 1.9 mmHg vs. 33.5 ± 1.8 mmHg, P < 0.001). The TEI index and E/E' did not change with hypoxia. Hyperoxia reduced strain rate in 10 left ventricular segments, global systolic contraction amplitude was decreased (8.83 ± 0.38, P < 0.001 vs. normoxia) while right ventricular function was unchanged. The spectral and tissue Doppler TEI indexes were significantly increased but E/E' did not change with hyperoxia.

**Conclusion:**

Hypoxia improves and hyperoxia worsens systolic myocardial performance in healthy male volunteers. Tissue Doppler measures of diastolic function are unaffected by hypoxia/hyperoxia which support that the changes in myocardial performance are secondary to changes in vascular tone. It remains to be settled whether oxygen therapy to patients with heart disease is a consistent rational treatment.

## Introduction

Low oxygen tension dilates coronary arteries and high oxygen tension is a coronary vasoconstrictor. Yet oxygen supplementation remains first line treatment in cardiovascular disease states such as myocardial infarction and pulmonary oedema.

Endothelium-dependent vasodilation is reduced in patients with ischemic heart disease [1] and such patients are believed to have a reduced ability to counteract the circulatory consequences of systemic (air line travel, high altitude stay) and regional (coronary artery stenosis) hypoxia. Spontaneous nocturnal hypoxia with desaturation for hours is a frequent phenomenon in patients with severe coronary artery disease [2]. Left atrial, left ventricular (LV), and right ventricular (RV) end-systolic diameter fall during simulated extreme altitude [3] and during moderate altitude exposure [4]. There is controversy concerning myocardial performance during hypoxia; improvement [4], no change [3,5,6]. and worsening [7] of left ventricular systolic function, have been described. One study looked at diastolic function expressed as E/A ratio and found a reduction with hypoxia [3].

Hyperoxia, a condition in which the total oxygen content of the body is increased above that normally existing at sea level, is associated with impairment of cardiac relaxation and increased left ventricular filling pressures in patients with and without congestive heart failure [8].

Tissue Doppler imaging (TDI) objectively derives measurements of contraction and relaxation velocities directly from the myocardium and thus yields information not previously accessible by echocardiography [9]. We used TDI to study myocardial performance in healthy volunteers and we hypothesized that hypoxia increases and hyperoxia reduces myocardial performance.

## Material and Methods

### Subjects

Seven healthy men, (age 25–46 (mean 38) years) completed the study. All subjects were normotensive, non-smokers, on no medication, had a normal left ventricular function by 2-D echocardiography, and had no family history of ischemic heart disease. The local ethical committee approved the study.

### Ventilation system

We used a system consisting of a ventilator, a gas analyser with pulse oximeter, and a computer. Computer programs control the experimental procedure and continuously collect data from the ventilator and gas analyser [10].

### Echocardiography

Echocardiography was performed from the apical acoustic window. Tissue Doppler recordings were acquired as digital loops in the apical 4-chamber, 2-chamber, and long-axis views [9]. To avoid aliasing, the settings of the ultrasound equipment and colour-coded area were adjusted to obtain the highest possible frame rate. We measured peak strain in the 16-segment left ventricular model and in 3 segments in the right. From the color-coded tissue tracking image, the motion amplitude toward the apex in systole was recorded in each segment. The global systolic contraction amplitude (GSCA) was calculated as the average shortening amplitude of all 16 segments. The peak E velocity was obtained by pulsed Doppler measurements of the mitral inflow at the tip of the mitral leaflets. We further assessed isovolumetric acceleration, TEI index (spectral and TDI), tricuspid regurgitation and pulmonary ejection time. The tissue E' velocity was obtained by Tissue Doppler at the lateral mitral annulus. E/E' has been proposed as a tool for assessing LV filling pressures that combines the influence of transmitral driving pressure and myocardial relaxation [11]. The TEI index (spectral or TDI), a combined measure of systolic and diastolic function, was assessed by Doppler time intervals from the mitral inflow and LV outflow tract or the time intervals were obtained by TDI at the lateral mitral annulus. The TEI index was calculated by a-b/b where the time interval "a" was measured from cessation to onset of mitral inflow and the time interval "b" was the duration of the LV outflow velocity profile. To minimize the variability of the measurements, all ECHO recordings were performed and analyzed in a blinded fashion by the same author (P.S.).

### Study protocol

TDI was performed when the subjects had rested for 15 minutes breathing room air (SpO_2 _97.9 ± 0.1 %), after 5 minutes of respiratory equilibrium during hypoxia (SpO_2 _77.6 ± 1.2 %), and after 5 minutes of respiratory equilibrium during hyperoxia (SpO_2 _99.0 ± 0.2 %). Heart rate was continuously recorded on computer by means of the pulse oximeter. Blood pressure was measured once during each of the three respiratory steady state situations by an automatic blood pressure measuring device based on the oscillometric method.

### Statistical analysis

All data are presented as mean ± SEM. Comparisons of the responses to changes in F_i_O_2 _(normoxia, hypoxia and hyperoxia) were made with a one-way repeated-measures analysis of variance. The Student-Newman-Keuls test was used post hoc to identify pairwise differences. Differences were considered statistically significant when P < 0.05.

## Results

### Respiratory parameters and hemodynamics

FiO_2 _and FeO_2 _decreased with hypoxia and increased with 100% oxygen breathing (table 1). FeCO_2 _and tidal volume were unchanged in all three test situations, reflecting that the subjects were in respiratory steady state. There was a small, but statistically significant *decrease *in respiratory rate from normoxia to hypoxia, which might reflect that the subjects were more accustomed to the test situation at this stage.

**Figure 1:**
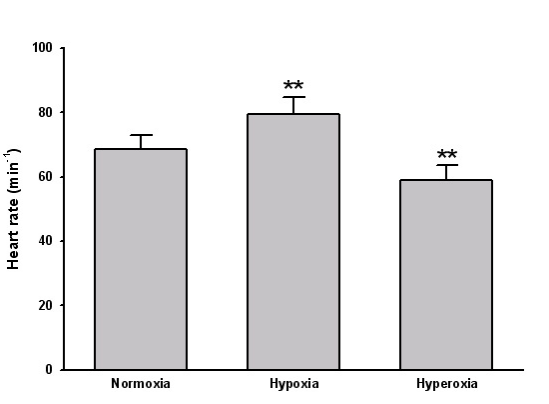
Bar graph depicting heart rate during normoxia, hypoxia and hyperoxia. ** P < 0.01 vs. normoxia.

There was a significant increase in heart rate with hypoxia and a decrease with hyperoxia (figure 1). Neither systolic (126 ± 6, 123 ± 8, 122 ± 12 mmHg, respectively, P = ns) nor diastolic blood pressure (80 ± 5, 78 ± 8, 81 ± 6 mmHg, respectively, P = ns) changed significantly with test situation.

### TDI and spectral echocardiography, hypoxia

**Figure 2:**
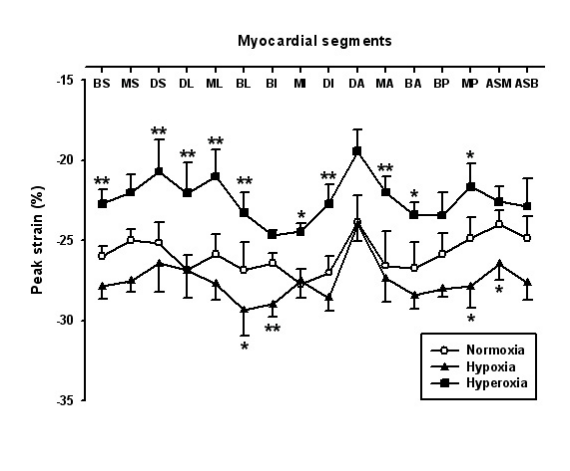
Comparison of peak systolic strain in the 16-segment left ventricular model illustrating the effects of hypoxia and hyperoxia. *A*, Anterior; *B*, basal; *D*, distal; *I*, inferior; *L*, lateral; *M*, mid; *P*, posterior; *S*, septal. * P < 0.05, ** P < 0.01 vs. normoxia.

**Figure 3:**
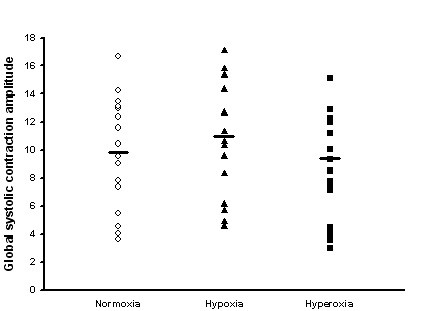
Tissue tracking score index on the basis of the 16-segment left ventricular model illustrating the effects of hypoxia and hyperoxia.

**Figure 4:**
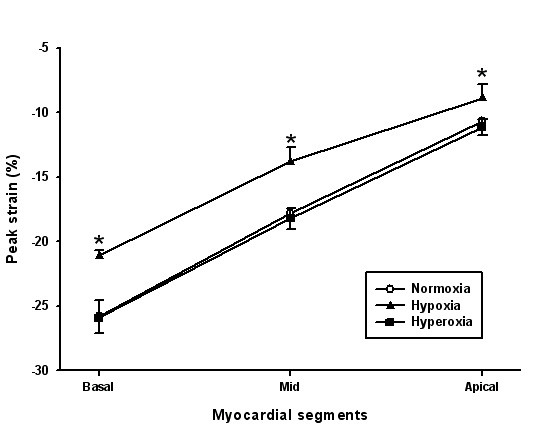
Comparison of peak systolic strain in the right ventricle model illustrating the effects of hypoxia and hyperoxia. * P < 0.05.

**Figure 5:**
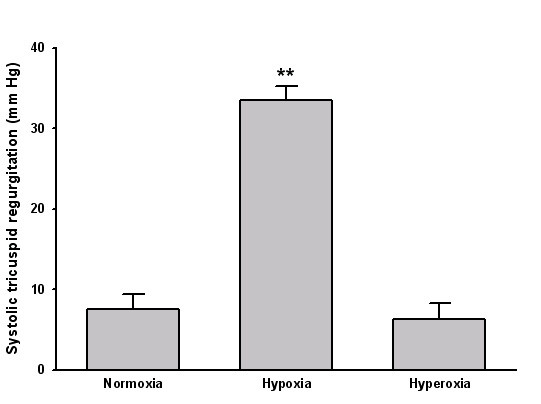
Bar graph depicting systolic tricuspid regurgitation during normoxia, hypoxia and hyperoxia. ** P < 0.01 vs. normoxia.

A significant increase in strain rate was found in 4 segments (figure 2). GSCA increased with hypoxia (9.76 ± 0.41 vs. 10.87 ± 0.42, P < 0.001, figure 3). Tissue tracking displacement was reduced in all three right ventricular segments (figure 4) and systolic tricuspid regurgitation increased with hypoxia (figure 5). The TEI index (spectral or TDI) and E/E' did not change with hypoxia.

### TDI and spectral echocardiography, hyperoxia

Hyperoxia worsened left ventricular function. Strain rate was reduced in 10 segments (figure 2) with preponderance in the lateral and anterior segments. GSCA was reduced (8.83 ± 0.38, P < 0.001 vs. normoxia). Tissue tracking displacement did not change in the right ventricular segments (figure 4) and systolic tricuspid regurgitation was unchanged compared with normoxia (figure 5)). The TDI TEI index was significantly increased with hyperoxia (0.32 ± 0.04 vs. 0.45 ± 0.05, P < 0.001) and the spectral TEI index showed similar changes. E/E' did not change with hyperoxia.

## Discussion

The main findings of the present study of healthy volunteers are: 1) hypoxia increased strain rate and tissue tracking displacement. 2) Hypoxia increased tricuspid regurgitation and reduced right ventricular tissue tracking displacement. 3) Hyperoxia reduced strain rate and tissue tracking displacement and increased the TEI index. The novelty of our findings, when compared to the literature, is the demonstration that longitudinal myocardial function, and thus the function of the subendocardium, is sensitive to moderate changes in inspired oxygen.

Patients with ischemic heart disease and heart failure frequently encounter hypoxia but the consequences of hypoxia on left ventricular function remain a matter of controversy, in part because of differences in methodology and the measured parameters. In early studies using roentgenkymograms [12] and dye injection [13] hypoxia was shown to increase cardiac output at rest despite reduced [13] or unchanged [12] stroke volume. The authors explained the increase in cardiac output by increased heart rate [12,13] Myocardial blood flow in the left and right ventricles increased at high altitude in dogs studied with radioactive microspheres [14] and in healthy controls using positron emission tomography [15]. In a study using M-mode echocardiography at high altitude [4] percent fractional shortening and velocity of circumferential fiber shortening remained normal while LV isovolumetric contraction time shortened. Echocardiography was also employed in a simulated ascent of Mount Everest [3] and an insignificant increase in fractional shortening and ejection fraction was found. It is generally accepted that right-sided pressures increase with hypoxia [3,14] We speculate that the improvement in tissue tracking displacement in our study reflects the systemic vasodilation which is another consequence of hypoxia [16]. On the other hand, RV-systolic function decreased which is likely to be correlated to the increased systolic pulmonary pressure reflecting the increased pulmonary vascular resistance during hypoxia.

Hemoglobin saturation in healthy persons increase very little from breathing room air to breathing 100% oxygen. Nevertheless, profound cardiovascular effects were found. This is probably because of an anticipated increase in oxygen dissolved into plasma from 0.32% to 2.09% [17]. Hyperoxia is a possible product of oxygen therapy when administered to patients with heart disease during acute illness. Hyperoxia reduces cardiac output as documented with roentgenkymograms [12], dye injection [18,19]., echocardiography [20] heart catheterisation [8] and indirectly by measurement of isometric systolic tension by means of strain gauge in dogs [21]. Reduced cardiac output with hyperoxia has even been demonstrated in patients with myocardial infarction using dye injection [22]. Some of the reduction in cardiac output may be explained by the observation that hyperoxia reduces heart rate as seen in our study and previously [19,23-25] Reduced heart rate is, however, not an entirely consistent finding [8,20,22,26] but in these four studies this could be because of high sympathetic tone (stay in a hyperbaric chamber [20], acute myocardial infarction [22], open heart surgery [26], heart catheterisation [8]). We found that heart rate increased with hypoxia and decreased with hyperoxia. This might have affected our measures of LV systolic and diastolic function but we consider this unlikely on the basis of our previous studies [11,27] demonstrating that TEI index, strain rate and the tissue tracking was unrelated to heart rate. During hypoxia as well as hyperoxia no change in LV filling pressure was noted as the E/E' ratio was unchanged. The individual parameters in the E/E' ratio are known to be influenced by heart rate but as a ratio it seems independent of heart rate and load conditions [11,27]. Therefore, we did not perform any adjustments for heart rate in the evaluation of LV diastolic function.

Because of the finding of no change [19] or a discrete rise [18,22] in blood pressure, a reduction in cardiac output results in an increased systemic vascular resistance during hyperoxia [18,19,22] Regardless of the fact that sympathetic tone may be affected by hyperoxia, even after complete sympathetic blockade myocardial contractile force remains reduced [26]. In the present study both tissue tracking displacement and strain rate worsened during hyperoxia. It seems plausible that this deterioration could be explained by systemic vasoconstriction [21] increasing afterload.

In conclusion, hypoxia improves and hyperoxia worsens systolic myocardial performance in healthy male volunteers. TDI measures of diastolic function are unaffected by hypoxia/hyperoxia which support that the changes in myocardial performance are secondary to changes in vascular tone. It remains to be settled whether oxygen therapy to patients with heart disease is a rational treatment that may sometimes be harmful or whether supplemental oxygen consistently results in an overall gain in delivered oxygen.
